# Quantitative Assessment of Parenchymal Involvement Using 3D Lung Model in Adolescent With Covid-19 Interstitial Pneumonia

**DOI:** 10.3389/fped.2020.00453

**Published:** 2020-08-05

**Authors:** Luca Borro, Paolo Ciliberti, Teresa Pia Santangelo, Andrea Magistrelli, Andrea Campana, Francesca Calò Carducci, Marabotto Caterina, Paolo Tomà, Aurelio Secinaro

**Affiliations:** ^1^Department of Imaging, Bambino Gesù Children's Hospital IRCSS, Rome, Italy; ^2^Pediatric Cardiology and Pediatric Cardiac Surgery Department, Bambino Gesù Children's Hospital IRCSS, Rome, Italy; ^3^Department of Pediatric Medicine, Bambino Gesù Children's Hospital, Rome, Italy; ^4^Pediatric University Hospital-Department, Bambino Gesù Children's Hospital, Rome, Italy; ^5^Unit of General Pediatrics and Pediatric Infectious Diseases, Bambino Gesù Children's Hospital, Rome, Italy

**Keywords:** 3D lung reconstructions, 3D modeling in pneumonia, 3D parenchima reconstruction, 3D rendering in pneumonia, 3D in Covid19, 3D quantify in pneumonia

## Abstract

**Background:** Amount of parenchymal involvement in patients with interstitial pneumonia Covid-19 related, seems to be associated with a worse prognosis. Nowadays 3D reconstruction imaging is expanding its role in clinical medical practice. We aimed to use 3D lung reconstruction of a young lady affected by Sars-CoV2 infection and interstitial pneumonia, to better visualize, and quantitatively assess the parenchymal involvement.

**Methods:** Volumetric Chest CT scan was performed in a 15 years old girl with interstitial lung pneumonia, Sars-CoV2 infection related. 3D modeling of the lungs, with differentiation of healthy and affected parenchymal area were obtained by using multiple software.

**Results:** 3D reconstruction imaging allowed us to quantify the lung parenchyma involved, Self-explaining 3D images, useful for the understanding, and discussion of the clinical case were also obtained.

**Conclusions:** Quantitative Assessment of Parenchymal Involvement Using 3D Lung Model in Covid-19 Infection is feasible and it provides information which could play a role in the management and risk stratification of these patients.

## Introduction

The amount of parenchymal involvement in patients with interstitial pneumonia Covid-19 related seems to be associated with a worse prognosis (1).

Nowadays 3D reconstruction imaging is expanding its role in clinical medicine (2–5).

We describe the use of 3D lung reconstruction of a young lady affected by Covid-19 infection and interstitial pneumonia, to quantitatively assess the lung parenchyma involved.

In March, 2020, a 15-year-old girl was admitted to our Emergency Department, with a 7-day history of fever and anosmia. She had been complaining exertional shortness of breath over the previous 2 days. At initial evaluation oxygen saturation was 97% in room air. Chest X-Ray showed no significant consolidation with perihilar infiltrates mainly on the left basal side.

Her pharyngeal swab Real-Time Polymerase Chain Reaction (RT-PCR) was positive for Sars-Cov2 infection.

As further investigation the patient underwent Chest-CT. Non-contrast spiral high isotropic resolution CT acquisition was performed. In the left lower lobe multiple shaded areas of increased density, Ground Glass Opacity (GGO) type, were observed. There were also subpleural parenchymal “rounded” consolidations. Similar changes were seen in the lingular segment. The CT pattern was reliably related to a viral pneumonia with exclusive left lung involvement (6). Experimental treatment with azithromycin and hydroxychloroquine was started, with progressive improvement of the clinical condition.

Using CT images we performed a 3D advanced segmentation and reconstruction of both lungs, in order to better delineate and quantitatively assess the amount of parenchymal abnormalities.

Written informed consent of the family, for use of imaging and clinical data for research purposes, was obtained.

## Methods

CT high spatial resolution images with an isotropic voxel size of 1 mm and a smooth filter were used for the segmentation. The dataset was uploaded on an advanced 3D post-processing platform “Mimics” (Materialize, Belgium) and three different segmentation masks were defined: parenchyma, GGO areas, and spot consolidations. Ground-glass opacity (GGO) has been defined as an area of blurry increased lung opacity where vessels and bronchial structures may still be seen, whilst in consolidation such structures are concealed. Most commonly, GGO areas suggest inflammatory, or infiltrative interstitial lung disorders, such as interstitial pneumonia. Consolidation conversely is a region of normally compressible lung tissue that has filled with liquid instead of air, and suggest an infiltrative or inflammatory process involving the entire lung tissue.

**Figure 1:**
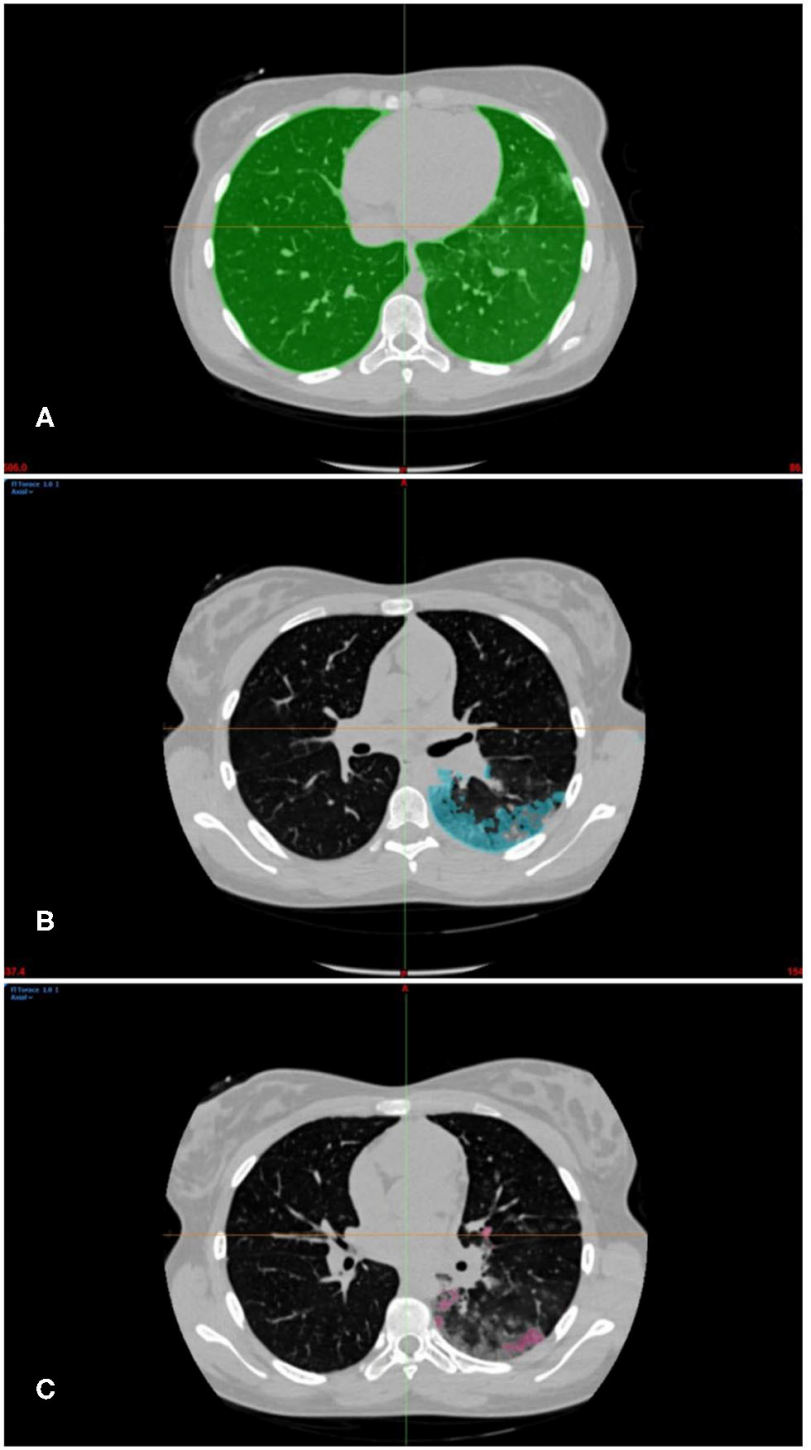
**(A)** Green mask of normal parenchyma segmentation within Hounsfield range −1024Hu to −543Hu. **(B)** Light blue mask of sub-pleural GGO areas segmentation within Hounsfield range between −957Hu to +1246Hu. **(C)** Pink mask spot consolidations segmentation within Hounsfield range between −834Hu to +356Hu.

Two semi-automatic reconstruction methods were applied. In particular, the thresholding algorithm, was an effective tool to segment normal lung parenchyma (7) (Figure 1A), whilst the region-growing algorithm was used for ground glass areas (Figure 1B) and spot consolidations (Figure 1C). In addition, some small areas of consolidation have been manually segmented.

We measured density values on healthy right lung with Hounsfield values from −1024Hu to −543Hu similar to typical range values of pediatric lungs (8).

Both GGO and parenchymal consolidations Hounsfield ranges were obtained by making several measurements on a different CT Slices (−957Hu to +1246Hu for GGO areas, and −834Hu to +356Hu for consolidations areas). Any transition areas between the two masks have been optimized with Boolean difference operations aimed at better defining the consolidations and GGO boundaries.

Each of the three masks has been 3D reconstructed with a specific mesh quality setup to obtain the highest resolution and the best 3D-2D correspondence.

In order to obtain an adequate 3D rendering and 3D mesh reconstruction, the model was optimized with the Rhinoceros software (McNeel, USA) to allow adequate 3D visualization for clinical purposes.

## Results

The 3D reconstructions allowed us to accurately calculate volumes of Lungs, GGO areas, and consolidation spots. A volume-relationship was then established between the different reconstructed parts, as shown in Table 1.

**Table 1:** Quantification of lung volumes obtained from CT 3D reconstructions.

	**Total lung**	**Left lung**	**Ground glass opacity**	**Consolidations spots**
Volume (ml)	4.724	2.136	240.94	26.30
Total lung Vol (%)	–	45.21	5.10	0.55
Left lung Vol (%)	–	–	11.28	1.23

Ground glass opacity areas were 5.1% of the entire lung volumes and 11.28% of the left lung. Consolidation spots represented 0.55% of the entire lung volumes and 1.23% of the left lung.

**Figure 2:**
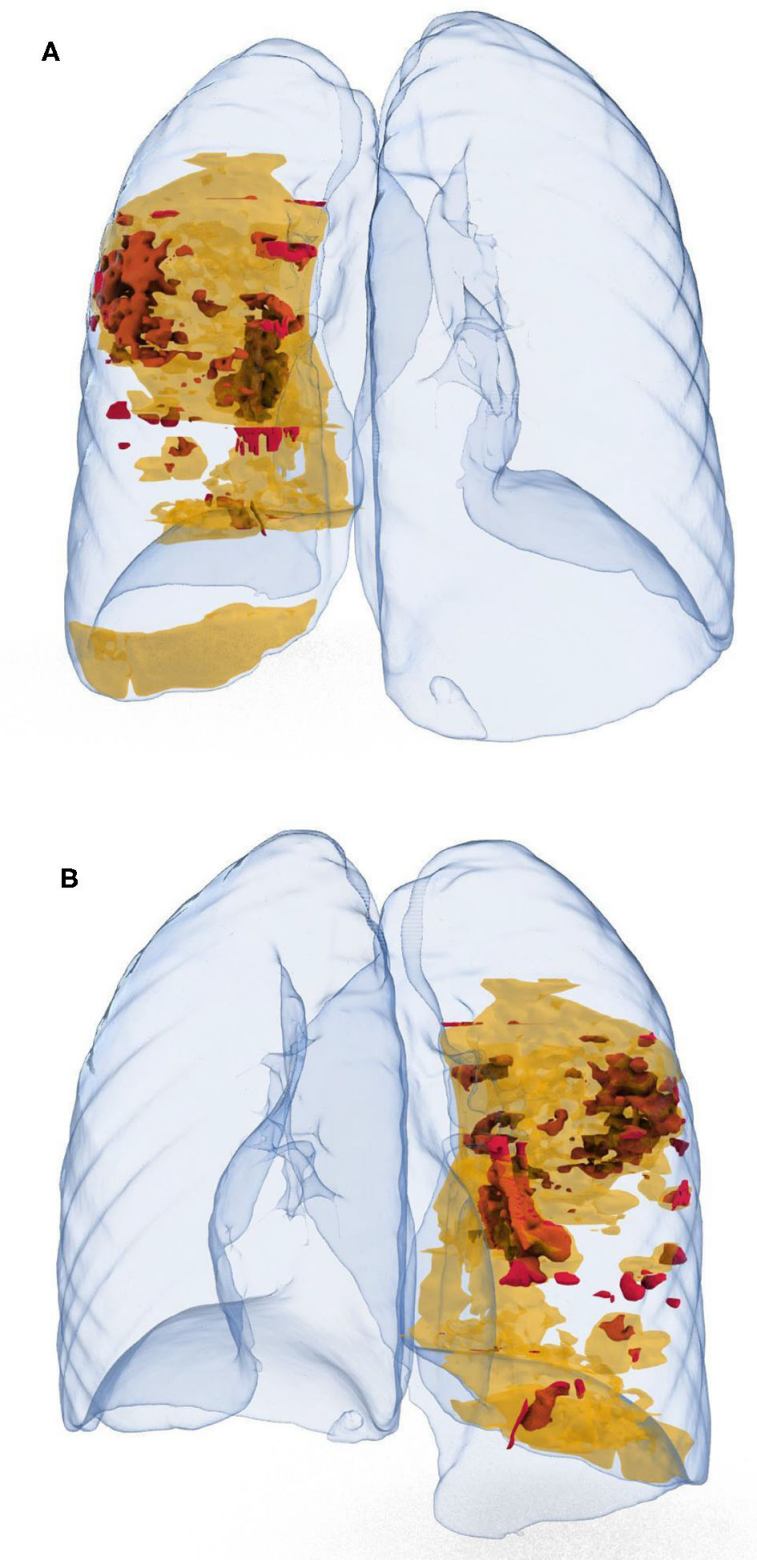
Back view **(A)**, and frontal view **(B)** of lungs parenchyma (crystal clear), with GGO areas in yellow, and spot consolidation in red.

3D rendering proved to be a very simple and effective approach for representing the lung anatomy including the distribution/topography of the different pathological areas. We obtained a series of different 3D volume rendering views to highlight radiological changes as shown in Figures 2A,B. We also produced a 3D movie (additional Video online) which shows a more comprehensive rotational rendering overview of the parenchymal changes, interstitial pneumonia related. The final rendering was uploaded on a 3D online viewer platform (Sketchfab), which allows the clinical team to explore and navigate the 3D model on a web-link, taking advantage of a very powerful rendering capability with no need for dedicated workstation and additional software.

## Discussion

3D modeling represents a “paradigm shift” from the classical descriptions of lung changes based on conventional radiological reformats to 3D volume rendering reconstructions, that could improve the understanding (and communication to families and patients) of the extent and nature of these lung changes. In addition, we try to test it as a potential tool for surveillance and comparisons in clinical practice and research activity.

In this case we apply advanced 3D segmentation tools to increase the diagnostic information in case of Covid-19 patient. In effect, the progressive impairment of lung tissues due to the Sars-Cov2 infection is one of the most threatening phenomenon for people affected by this viral infection (1). CT has been introduced in many diagnostic algorhytm to manage these patients in order to predict or anticipate severe clinical deterioration (9, 10). Detailed extent and quantification of affected lung volume, such as GGO or consolidation areas, could be helpful in the management of this pandemic infection. Therefore, accurate quantitative assessment might be useful and included in the CT scan report.

The 3D reconstruction and rendering views in addition to provide a comprehensive and self explaining lung “tissue characterization” with distribution of the different pathological areas, seems to be able to allow reliable lung volumes calculation with detailed analysis of the compromised areas.

We described our algorithm to obtain 3D rendering views and accurate lung volume calculations; it appears to be easily obtainable using a commercial 3D software and potentially reproducible even by using different 3D platforms. In addition, 3D visualization of lung lesions can provide to the medical staff a wider and self-explaining understanding of the underlying lung pathology of Covid-19 patients. As already reported (11) 3D models can also be used for communication with families and patients and for teacing purposes.

Moreover, since CT represents an important imaging biomarker and is pivotal to guide pharmacological management and improve ventilation strategies, further implementation of semi-automatic and/or fully automatic (AI-based) algorithms for image processing (12, 13) might be beneficial in order to rapidly and systematically provide accurate data about the extent of lung disease in these patients.

Main limitation of our study is that it is a single case investigation. Therefore, we can't assess its reproducibility, and its real impact on clinical practice. Our aim is to point out the opportunity and feasibility of this approach in the particular clinical setting of Covid-19 pandemic in order to stimulate its wider use. Further analysis aimed to assess the clinical impact for the management and risk stratification of this novel tool, and its reproducibility in this setting are obviously needed.

## Data Availability Statement

All datasets presented in this study are included in the article/Supplementary Material.

## Ethics Statement

Written informed consent was obtained from the legal guardian/next of kin of the participant for the publication of this case report and any identifying images or information.

## Author Contributions

LB and AS: idea for the article. AM: CT-exam. LB: images and video production. AM, AC, FC, MC, and PT: revision and approval of the manuscript. LB, AS, and PC: writing the article. All authors contributed to the article and approved the submitted version.

## Conflict of Interest

The authors declare that the research was conducted in the absence of any commercial or financial relationships that could be construed as a potential conflict of interest. The reviewer KM declared a past co-authorship with several of the authors AS and TS to the handling editor.
